# The effectiveness of cognitive behavioral therapy-based interventions for depression and anxiety in people living with HIV in low- and middle-income countries: A systematic review and meta-analysis

**DOI:** 10.1017/gmh.2026.10223

**Published:** 2026-05-13

**Authors:** Huma Mughal Azeemi, Monica Magadi, Saeed Farooq, Mukhtar Ul Haq Azeemi, Aliya Durrani, Muhammad Asim, Saima Aleem, Nadia Corp, Mirrat Gul, Ghasem Yadegarfar, James A. Prior

**Affiliations:** 1 School of Medicine, Keele University, UK; 2 Psychiatry, Lady Reading Hospital, Pakistan; 3 Institute of Public Health, Khyber Medical University, Pakistan; 4 Psychiatry, Mayo Hospital, Pakistan

**Keywords:** cognitive-behavioral therapy, HIV, depression, low- and middle-income countries, meta-analysis

## Abstract

Cognitive-behavioral therapy (CBT) has shown promising results in improving mental health outcomes among people living with human immunodeficiency virus (PLHIV) in low- and middle-income countries (LMICs), although findings remain mixed. This systematic review examined the effectiveness of CBT and CBT-based interventions in reducing depression and anxiety among PLHIV in LMICs and explored the CBT techniques used across studies. Randomized and non-randomized controlled trials were included, and standardized mean differences were pooled using random-effects meta-analysis. Eight studies focusing on depression were included in the meta-analysis. Results showed that CBT-based interventions produced a large positive effect on depression scores compared to treatment as usual (*g* = −0.85; 95% CI: −1.25 to −0.45). Several CBT components were compared, including therapy type, therapy provider, number of sessions, and session duration. Standard CBT showed greater effectiveness than CBT-based interventions (−1.18 vs. −0.42). CBT delivered by mental health professionals also had stronger effects on depression outcomes than interventions provided by non-mental health professionals (−1.18 vs. −0.37). Meta-analysis for anxiety outcomes could not be conducted because of limited available data.

## Graphical abstract

**Figure:**
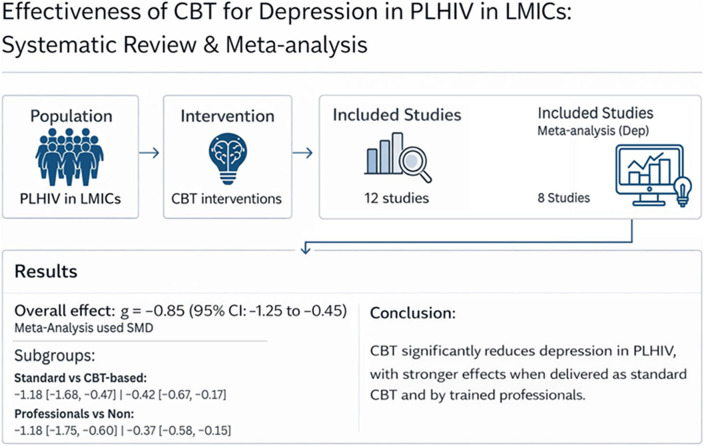

## Impact statement

This systematic review and meta-analysis deliver the most comprehensive synthesis to date on the effectiveness of CBT-based interventions for depression among PLHIV, with a particular focus on LMICS. Despite the well-documented link between HIV and common mental disorders (most commonly, depression, and anxiety), access to psychosocial interventions remains limited across LMICS. Our findings highlight a significant and clinically meaningful reduction in depressive symptoms following CBT-based interventions, underscoring the urgent need to develop culturally based CBT interventions which can be integrated into mental health care and HIV treatment frameworks. These results offer robust, evidence-based guidance for policymakers and health systems planners aiming to address the mental health treatment gap among PLHIV in resource-constrained settings.

## Introduction

Human Immunodeficiency Virus (HIV) attacks the body’s immune system, specifically targeting CD4 cells (CD4 T lymphocytes), which are crucial for defending against infections and diseases. Acquired Immunodeficiency Syndrome (AIDS) is an advanced stage of HIV (WHO, 2025).

The relationship between HIV/AIDS and mental health is closely intertwined. The global prevalence of anxiety and depression in people living with human immunodeficiency virus (PLHIV) has been reported to be 20% and 33%, respectively (Hoare et al., 2021). However, a higher prevalence of depression is apparent in developing/underdeveloped countries compared to developed ones (South America 44%, Asia 36%, Africa 31%–38%, Europe 22%). The impact on anxiety in this group by economic status remains unclear (Rezaei et al., 2019). A previous systematic review also reported that mental health conditions among PLHIV in low- and middle-income countries (LMICs) are notably higher than in their general population (Nakimuli-Mpungu et al., 2021).

A multi-region synthesis of depression in PLHIV noted prevalences of 22%–32% in the U.S. (High-Income Country (HIC) vs. 29%–48% in East Africa (LMIC)), suggesting a higher burden in LMIC (Adedeji et al., 2023; Hu et al., 2025; Tadesse et al., 2025). In Pakistan, Ahmed et al. found depression to range from 28% to 52%, and that anxiety was as high as 80% among PLHIV (Ahmed et al., 2021).

Despite the high prevalence of these mental health conditions, they remain underdiagnosed and undertreated, significantly impacting the quality of life and overall well-being of PLHIV (Chibanda et al., 2011; Tadesse et al., 2025). Since mental health problems lie along a continuum that extends from mild distress to persistent and severe symptoms, treatment for such conditions is crucial (Patel et al., 2018).

Cognitive-behavioral therapy (CBT), a form of talking therapy, can help a person manage their problems by changing the way they think, feel, and behave (Judieth, 2025). Standard CBT is a structured, time-limited, and manualized intervention that adheres to a defined theoretical model, typically involving techniques such as cognitive restructuring, behavioral experiments, problem-solving, and activity scheduling (Davies J Davies, 2025). In contrast, many contemporary CBT-based interventions adopt a more eclectic or integrative approach, incorporating elements from multiple therapeutic traditions. These interventions not only maintain some core features of CBT, such as focus on the interplay between thoughts, emotions, and behaviors, but also draw on techniques and philosophies from other schools of psychotherapy (Abdollahpour et al., 2022). Such integrative interventions are often tailored to specific populations or clinical contexts (Kabat-Zinn, 1990; Segal et al., 2002; Young et al., 2003).

Previous systematic reviews on the use of CBT among PLHIV have largely focused on its effectiveness for depression in HICs, which limits their generalizability to LMIC settings and to other common conditions such as anxiety (Gebru et al., 2024). Early reviews by Crepaz et al. and Spies et al. focused on the effectiveness of CBT as an intervention for depression and anxiety in PLHIV globally, but primarily identified studies in HIC (just a single study from LMIC in each) (Crepaz et al., 2008; Spies et al., 2013). Both reviews subsequently emphasized the need for more evidence from LMICs, particularly in sub-Saharan Africa and South Asia. Though the review by Van Lunen et al. did address anxiety as well as depressive outcomes, there remained limited representation for LMICs (van Luenen et al., 2018). A more recent review by Qin et al. in 2022 included both high- and middle-income settings but only had a small number of studies from resource-limited contexts (*n* = 8) and predominantly focused on depression outcomes. Finally, the review by Zeying et al. provided a general overview of psychosocial interventions, yet lacked specificity in their focus of intervention (CBT) and only partially explored outcomes, primarily concentrating on depression rather than anxiety (Du Zeying et al., 2022). Moreover, this review lacked a sensitivity analysis between the two, failing to discern nuanced differences in outcomes between LMICs and HICs. The predominance of previous original research and summary reviews from HICs constrains the transferability and relevance of findings to LMIC settings (Abdollahpour et al., 2019, Abdollahpour et al., 2018).

Findings from HICs, while informative, may have limited transferability to LMICs due to substantial differences in health system capacity, sociocultural contexts, and resource availability (Patel et al., 2018; WHO, 2018). With this area, it is also important to understand how well the different components and design choices of the CBT intervention function are in practice. This includes examining not only the content of the intervention but also how it is delivered, adapted, and received in real-world settings. Such insight is particularly relevant in LMICs, where mental health interventions often rely on task-sharing approaches and are delivered by various healthcare providers with differing levels of training and experience due to severe shortages of mental health professionals (Chibanda et al., 2011; Padmanathan and De Silva, 2013). The psychological treatments delivered specifically by non-specialist health workers have not previously been systematically evaluated.

Our aim was to identify and synthesize evidence on the effectiveness of CBT-based interventions for treating depression and anxiety among PLHIV in LMICs. The objectives were (1) to evaluate the effectiveness of CBT-based interventions in reducing symptoms of depression and anxiety among PLHIV and (2) to identify and analyze the components and characteristics of CBT-based interventions used for this population.

## Methods

A systematic review was conducted to identify studies using standard CBT or integrative CBT-based interventions for either the treatment of depression or anxiety in PLHIV in LMICs. Primary outcomes related to the level of depression or anxiety at study follow-up were extracted, synthesized, and (where possible) included in meta-analysis. Pooled outcomes underwent sensitivity analysis and were examined as groups based on the CBT design components to discern the effectiveness of such interventions. The review protocol was registered with PROSPERO (CRD42024485690).

### Search strategy

A systematic search was conducted using Medline, PsycINFO, EMBASE (OVID), CINAHL, and the Cochrane Central Register of Controlled Trials (CENTRAL) via the EBSCO platform. The search covered literature from 1980 to February 2025. Search strings were developed using the PICOS framework (Supplementary Table 1).

### Screening and selection process

Studies were included if they met the following PICOS-based criteria. Population: Adults (≥18 years), either existing or newly diagnosed (based on clinical tests (i.e., Viral load) or clinical records) with HIV/AIDS and currently being treated for depression or anxiety. Study populations were also required to be from LMICs. We used the LMIC status defined by the World Bank (2024–2025) according to the country income classification for study data collection. Countries categorized as low income, lower middle income, or upper middle income based on gross national income per capita calculated using the Atlas method were considered LMICs (Samuel Kofi Teeth Baah and Serajuddin, 2024). Intervention: Individual or group CBT-based treatments. Comparators: Control groups could include those receiving treatment as usual, on a waiting list, or receiving an attention placebo (a control condition where participants receive the same amount of therapist/researcher time, attention, and contact as the experimental group, but without the active therapeutic components of the intervention). Outcomes: post-intervention values, in mean depression and anxiety symptom score. Study Design: Both randomized and non-randomized controlled trials to provide a comprehensive synthesis of the available evidence. In some settings, particularly LMICs, randomized allocation is not always feasible, making non-randomized designs an important source of evidence in LMICs.

Two reviewers (MA and SA) independently evaluated the titles and abstracts of all identified studies using the predefined inclusion criteria. Full-text screening was conducted by the lead author (HM), while discrepancies during screening were addressed through discussion with both reviewers.

### Quality assessment

The Cochrane Risk of Bias (ROB-1) tool was applied to randomized controlled trials (Higgins et al., 2019). Studies were rated as “high risk” if at least one domain was rated high, “unclear risk” if all domains were unclear, and “low risk” if all domains were low. Studies with more domains assessed as “unclear risk” or a mix of “low” and “unclear” risk without any “high risk” domains were considered at an unclear overall risk of bias (Higgins 2023).

The ROBINS-I was used to assess methodological quality in non-randomized controlled trials (Sterne et al., 2016). Each study was independently evaluated across the seven ROBINS-I domains. For each domain, studies were judged as “low risk of bias” when they were considered comparable to a well-conducted randomized controlled trial. A “moderate risk of bias” was assigned when some methodological limitations were present but were unlikely to substantially distort the study findings. Studies were rated as having a “serious risk of bias” when important methodological problems were identified that could meaningfully affect the validity of the results. A “critical risk of bias” was assigned when methodological shortcomings were so severe that the results were considered unreliable. Where insufficient information was provided to permit a clear judgment, the domain was rated as “no information.”

The Template for Intervention Description and Replication (TIDieR) checklist was used to improve the reporting of interventions included in this review and make it easier for authors to structure accounts of their interventions. Evidence of publication bias was assessed using funnel plots and Egger’s test (Egger et al., 1997). A *p*-value of less than 0.05 was considered indicative of significant publication bias.

### Data extraction and synthesis

Extracted data included study methods, sample characteristics, intervention characteristics, and outcome data relating to depression and anxiety. This latter factor was the primary outcome of interest and consisted of post-intervention values, in depression or anxiety symptom scores. Study characteristics and outcomes were narratively summarized and presented in tables, including descriptive statistics (e.g., means, standard deviations (SDs)) and effect sizes with 95% confidence intervals (95% CI). Interventions were grouped by mode of delivery, and their components were tabulated to facilitate comparisons.

A random-effects meta-analysis of standardized mean differences (SMDs) was initially conducted to estimate the pooled effectiveness of CBT-based interventions (Stata 2007, Version 17.0). Subgroup meta-analyses of reported intervention characteristics (e.g., CBT components, type, or delivery mode), therapy provider (e.g., psychologist or non-specialist), techniques (e.g., cognitive restructuring, stress management, or problem-solving), and studied design factors were also carried out where possible.

Depression outcomes were reported at varying time points across included studies, including immediate post-treatment and multiple follow-up periods. Our primary objective was to estimate the overall effect of the intervention on depressive symptoms, rather than effects at a single, fixed follow-up time. Pooling outcomes across time points, therefore, allowed us to synthesize the maximum available evidence and avoid unnecessary exclusion of studies that did not report outcomes at identical time points.

Where multiple time points were reported within a single study, only one time point per study was included in the meta-analysis to avoid unit-of-analysis errors. The selected time point was the most comparable, consistent, and clinically relevant outcome, prioritizing post-treatment measures at 3 months, 6 months, and 12 months. Some studies included follow-up periods of up to three years; however, only the post-intervention outcomes at the 12-month follow-up were used in this review (Guo et al., 2020). This approach is consistent with guidance from the Cochrane Handbook (Higgins et al., 2011). When follow-up times vary across studies and a common time point is not consistently reported, although post-treatment outcomes were available in several studies, they were not uniformly reported across all included studies. Restricting the analysis to post-treatment outcomes alone would have resulted in the exclusion of relevant studies and reduced the statistical power and generalizability of the findings.

## Results

**Figure 1.**
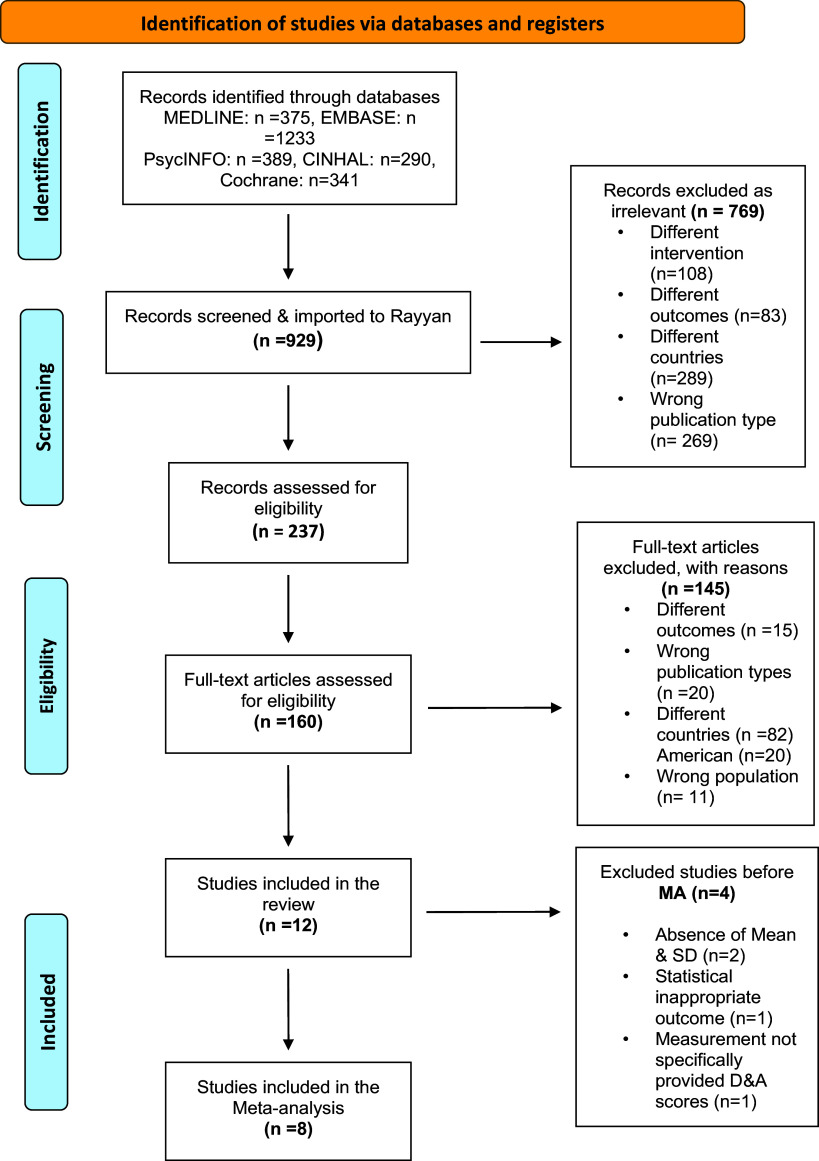
PRISMA flow diagram. Figure 1. long description.

The systematic search identified 2,727 studies, and after duplicate removal using the review management software Rayyan, 929 studies remained. After title and abstract screening, 769 studies were excluded, resulting in 160 studies eligible for full-text review (Figure 1). During the full-text screening, 148 studies were excluded based on predefined criteria, leaving 12 studies for inclusion in the systematic review. Of these, eight were included in the meta-analysis, with four not providing the necessary data for the meta-analysis (Pujiati and Gasem, 2022; Haas et al., 2023; Hemmati Sabet et al., 2013; Nwobi et al., 2018).

### Characteristics of included studies

Of the 12 included studies, seven included both men and women (Chattopadhyay et al., 2017; Nwobi et al., 2018; Guo et al., 2020; Safren et al., 2021; Pujiati and Gasem, 2022; Abbas et al., 2023; Haas et al., 2023), three focused exclusively on males (Hemmati Sabet et al., 2013; Atefeh Nobakht A et al., 2018; Jalali et al., 2019), and two just on females (Chibanda et al., 2011; Kaaya et al., 2022). The participants’ ages ranged from 18 to 63 years (Table 1).

**Table 1.** Characteristics of the included studies Table 1. long description.

Author/ year	Country	N	Study design	Age	Male (%)	HIV diagnosis	Depression/ Anxiety (D&A)	Intervention (*I*), no of participants in intervention (NI)	Control group
Abbas et al. (2023)	Pakistan	126	RCT	**Range** 20–55 **Mean (SD)** 37.5 (10.11)	52 41%	Registered with the ART clinic and receiving ART regularly	PHQ-9	*I* = B-CBT NI = 63	Wait-list control (WLC) *N* = 63
Chattopadhyay et al. (2017)	India	60	RCT	**Range** 20–60 **Mean** 40 (11.55)	39 (65%)	All cases of HIV attending the referral center. The CD4 lymphocyte count was measured at baseline	BDI	*I* = CBT NI = 30	TAU (30) Psychoeducation and supportive counseling with continuation of ART
Nobakht A et al. (2018)	Iran	288	RCT	**Mean** 30 (5)	100%	HIV-positive women referring to Imam Khomeini Hospital Consultation Centre	H = already-diagnosed HIV-positive cases. DDA = DASS-21	*I* = CBT NI = 14	TAU (*N* = 14)
Safren et al. (2021)	South Africa	163	RCT	NR	48 29.8%	Underwent blood draw for HIV RNA viral load and CD4 from medical records. HIV RNA viral load >400 copies/ml after first-line treatment	Using MDD module of the MINI.	*I* = CBT-AD NI = 80	Enhanced TAU = (81)
Hemmati Sabet et al. (2013)	Iran	42	Semi-experimental study, pre-post design	NR	100%	Diagnosed HIV-positive men	16 > in anxiety scores on the questionnaires of D&A, & stress scale	*I* = CBT NI = 21	Group training of stress management = 21
Nwobi et al. (2018)	Nigeria	28	RCT	**Mean** *I* = 45.71 ± 3.31 *C* = 43.57 ± 3.63	*I* = 5 41.7%, *C* = 7 58.3%	Already-diagnosed HIV patients	HADS	*I* = CBSM NI = 14	No treatment = 14
Guo et al. (2020)	Guangzhou, China	300	RCT	≥18 years **Mean** 28 (5.8)	277 92%	HIV seropositive	CES-D scale score ≥ 16	*I* = CBSM	WeChat platform
Chibanda (2014)	Zimbabwe	58	RCT	**Mean** 25	0%	NR	DSM-IV	*I* = PST NI = (30)	TAU (28), Amitriptyline (50 mg)
Kaaya et al. (2022)	Tanzania	742	Cluster-randomized controlled trial (RCT).	**Mean** *I* = 29.8 (5.5) *C* = 29.5 (5.3)	0%	HIV-positive pregnant women were registered in the year prior to study start	DD = PHQ-9	*I* = Healthy options including PST & CBT components plus EUDC NI = 395	EUDC mhGap treatment guidelines (347)
Haas et al., (2023)	Zimbabwe	516	RCT - open-label pragmatic cluster trial	**Mean** 45.6	78 15.1%	Already-diagnosed with HIV at ART clinics	AD = SSQ-14 DD = PHQ-9	*I* = "Friendship Bench. NI = "244 (across 8 clusters), .	ESC Intervention = 272 (across 8 clusters) counseling, education, medication management, and referral to specialized services
Pujiati et al. (2023)	Indonesia	62	RCT	**Mean** *I* = 35.10 ± 6.01 *C* = 33.35 ± 7.06	(54.2%)	PLHIV on ART diagnosed with HIV.	Diagnosis of depression in HIV patients was performed by a psychiatrist	*I* = MBCT +Hypnosis NI = 31	MBCT 31
Jalali et al. (2019)	Iran	42	Between-groups pre-posttest.	**Mean** *I* = 32.60 ± 4.95 *C* = 30.96 ± 3.35	42 100%	Male prisoners living with HIV	Clinical interviews, score > 14 on BDI	*I* = CSF NI = 21	WLC group 21, received therapy afterwards

BDI = Beck Depression Inventory, BDI-IA = Beck Depression Inventory-IA, CBSM = Cognitive-Behavioral Stress Management, MBCT = Mindfulness-Based Cognitive Therapy, CSF = Cognitive schema-focused, PST = Problem-solving therapy, CES-D = Center for Epidemiologic Studies-Depression Scale, DASS-21 = Depression Anxiety and Stress Scales, DSM-IV = Diagnostic and Statistical Manual of Mental Disorders (Fourth Edition), EPDS = Edinburgh postnatal depression scale, GMAS = General Medication Adherence Scale, HADS = Hospital Anxiety and Depression Scale, HSS = HIV Stigma Scale, HRQOL = Health related Quality of Life, MBSR = Mindfulness-based stress management, MCBT = Mindfulness-Based Cognitive Therapy, MDD = major depressive disorder, MINI = Mini International Neuropsychiatric Interview, MSCL = Medical Symptom Checklist, PLHIV = people with human immunodeficiency virus, SQ-SF = Schema Questionnaire–Short Form, SSQ = Shona Symptoms Questionnaire, SF-36 = Short-form 36, VCT = Voluntary Counseling and Testing, ESC = Enhanced standard care, EUDC = Enhanced usual care for depression, WLC = Waiting list control.

Three studies were conducted in Iran (Hemmati Sabet et al., 2013; Nobakht A et al., 2018; Jalali et al., 2019) and two studies were carried out in Zimbabwe (Chibanda et al., 2014; Haas et al., 2023). Single studies were conducted in China (Guo et al., 2020), Central Africa (Safren et al., 2021), Pakistan (Abbas et al., 2023), India (Chattopadhyay et al., 2017), Indonesia (Pujiati and Gasem 2022), Tanzania (Kaaya et al., 2022), and Nigeria (Nwobi et al., 2018) (Table 1).

Healthcare settings were varied. Two studies were delivered in anti-retroviral therapy (ART) clinics (Safren et al., 2021; Abbas et al., 2023), one was in an HIV outpatient clinic (Guo et al., 2020), two in tertiary-care hospitals (Chattopadhyay et al., 2017; Nwobi et al., 2018), and one in a primary care urban clinic (Chibanda et al., 2014). One was carried out in the 16 government-managed antenatal care clinics that provided HIV care for pregnant women, one in rural health facilities (Kaaya et al., 2022; Haas et al., 2023), two in health consultant clinics (Hemmati Sabet et al., 2013; Nobakht et al., 2018), one in hospital-based consultation centers and one in positive clubs providing psychological and social support (Jalali et al., 2019; Pujiati and Gasem, 2022) (Table 1).

The included studies had recruited patients with HIV through clinical diagnosis, by Drs, HIV tests, positive viral load, and some studies reported the CD4 cell count ranges, and also patients who are on the ART (Table 1). Regarding the identification of the presence of depression or anxiety within the samples, two studies utilized the Diagnostic & Statistical Manual for Mental Disorders (DSM) diagnostic criteria and clinical interviews for depression and anxiety assessment, while 10 studies used different psychological scales for diagnosis and measuring the severity of depression using scales, that is, PHQ-9 or HAM-D, alongside interviews for diagnosis.

The studies examined a variety of intervention types. Five studies focused exclusively on standard CBT (Hemmati Sabet et al., 2013; Chattopadhyay et al., 2017; Nobakht et al., 2018; Abbas et al., 2023) and Safren et al. on CBT-Adherence (CBT-AD) (Safren et al., 2021), while the remaining seven studies utilized interventions based on CBT principles using a mix of approaches (Chibanda et al., 2014; Nwobi et al., 2018; Jalali et al., 2019; Guo et al., 2020; Kaaya et al., 2022; Pujiati and Gasem, 2022; Haas et al., 2023). Group interventions were reported in eight studies, while four focused on individual-based interventions. In terms of study design, 10 were randomized controlled trials (RCTs), of which two were cluster RCTs, and two were non-randomized controlled trials using pre- and post-measures.

Depression was assessed as a primary outcome in all 12 studies. Various measures were used to define depressive symptoms, including the PHQ-9, in three studies (Kaaya et al., 2022; Abbas et al., 2023; Haas et al., 2023), the Beck Depression Inventory (BDI) (Beck, 1996) in two studies (Chattopadhyay et al., 2017; Jalali et al., 2019), the Centre for epidemiological studies-depression (CES-D) scale (Guo et al., 2020), the Edinburgh Postnatal Depression Scale (EPDS), and the Hamilton Depression Rating Scale (HAM-D) were all only used in a single study (Safren et al., 2012) (Table 2).

**Table 2.** Outcomes of depression Table 2. long description.

		Intervention group						Control group		
Lead author & year	Country	n	Outcome measure	Length of follow-up (months)	*N* added in the analysis	Baseline mean (SD)	Post Mean (SD)	n	Baseline mean (SD)	Post mean (SD)
Abbas et al. (2023)	Pakistan	63	PHQ-9	2	80	11.70(4.23)	3.10(2.79)	63	9.92(3.42)	9.62(3.20)
Chattopadhyay et al. (2017)	India	30	BDI	6	57	20.53 (9.71)	15.40 (7.00)	30	21.33 (9.45)	23.37 (10.14)
			Adherence			89.31 (2.97)	100.00 (0.00)		88.82 (5.10)	97.51 (3.03)
Nobakht A et al. (2018)	Iran	30	DASS-D	1	60	31.1 ± 11.9	24.9 ± 10	30	36.1 ± 12.1	35.1 ± 11.4
Safren et al. (2021)	South Africa	80	HAM-D	12	161	23.75 (0.83)	7.51 (0.92)	81	25.76 (0.83)	13.15 (0.94)
			Adherence using electronic monitoring (wise pill device)							
Hemmati Sabet et al., (2013)	Iran	16	DASS-D	NR	NR	29.07 (6.181)	23.73 (4.949)	16	6.038 (27.4)	4.926 (28)
Nwobi et al. (2018)	Nigeria	14	HADS	3	28	38.29 (1.33)	12.57 (1.69)	14	37.86 (1.23)	38.36 (1.22)
Guo et al. (2020)	China	150	CES-D	12	300	23.9 (6.4)	18.1 (11.3)	150	24.3 (6.9)	23.3 (12.4)
			PHQ-9	12		10.2 (4.5)	7.2 (5.1)	150	10.7 (5.1)	8.5 (5.5)
Chibanda et al. (20114)	Zimbabwe	30	EPDS	1.5	58	17.3 + 3.7	8.22 + 3.6	28	17.9 + 3.9	10.7 + 2.7
Kaaya et al. (2022)	Tanzania	353	PHQ-9	9	742	11.3 (3.2)	2.6 (3.7)	288	11.6 (3)	3.6 (3.4)
Haas et al. (2023)	Zimbabwe	244	PHQ-9	12	460	8.3 (3.6)	NA	272	7.2 (3.2)	NA
Pujiati et al. (2023)	Indonesia	31				35.0 ± 5.1	22.9 ± 5.4	31	34.3 ± 4.8	29.3 ± 4.9
Jalili et al. (2019)	Iran	21	BDI	NR	NR	25.04 (7.22)	12.14 (6.52)	21	18.14 (9.32)	19.57 (8.36)

### Summary of depression outcomes

Eight included studies reported mean and SD values across various outcome measures to evaluate the efficacy of standard CBT and CBT-based interventions in reducing depression/depressive symptoms among PLHIV (Chibanda et al., 2015; Chattopadhyay et al., 2017; Nobakht et al., 2018; Jalali et al., 2019; Guo et al., 2020; Safren et al., 2021; Kaaya et al., 2022; Abbas et al., 2023).

### Standard CBT interventions

Five studies evaluated standard CBT. Abbas et al. (2023) reported significantly lower post-intervention depression scores in the CBT group compared with the wait-list control (WLC) group at two months. Similarly, Chattopadhyay et al. (2017) found significant improvement in the CBT group relative to treatment as usual (TAU) alongside improved treatment adherence over six months. Safren et al. (2021) demonstrated that CBT for adherence and depression (CBT-AD) resulted in a 4.88-point greater reduction in depression scores over 12 months compared with enhanced treatment as usual (ETAU), with mean scores in the CBT group versus in the ETAU group (*p* = 0.0016). Nobakht et al. (2018) also reported a statistically significant reduction in depression scores in the CBT group from baseline to post-intervention compared with minimal change in the control group post-intervention.

### CBT-based interventions

CBT-based interventions similarly demonstrated beneficial effects. Kaaya et al. (2022) reported an adjusted mean difference of −1.03 (*p* < 0.05) for a combined CBT and problem-solving therapy (PST) intervention, with a mean depression score compared with enhanced usual care. Chibanda et al. (2011) found significant reductions in postnatal depression among HIV-positive women receiving PST, with mean scores compared to TAU group receiving amitriptyline.

Guo et al. (2020) demonstrated that CBT-based stress management significantly reduced depression and stress levels, with the intervention group showing a decrease from baseline to post-intervention compared with smaller reductions in the control group. Jalali et al. (2019) reported that participants receiving cognitive schema-focused therapy had significantly lower depression scores than those in the WLC group.

Two studies were excluded from meta-analysis due to limitations in data reporting. Hemmati Sabet et al. (2013) reported substantial reductions in depression scores on the DASS-D in both the CBT group and the control group. However, inconsistencies in the reporting of SD values precluded inclusion in the meta-analysis. Haas et al. (2023), which evaluated a CBT-based Friendship Bench intervention among 516 participants, reported baseline PHQ-9 scores in the intervention group and in the control group, with a 12-month mean difference of 0.74 (95% CI: −0.60 to 2.08). As post-intervention mean and SD values were not reported, this study could not be included in the meta-analysis.

### Summary of anxiety outcomes

Anxiety was assessed in four studies, with two determining anxiety using the Depression, Anxiety, and Stress Scale (DASS-21) (Hemmati Sabet et al., 2013; Nobakht A et al., 2018). Nobakht A et al. (2018) reported baseline scores of the intervention group on DASS 30.2 (SD 10) and follow-up scores, 22.7 (SD 8.1), compared to the control group from baseline 34.5 (SD 10.4) to follow-up 33 (SD 8) (Atefeh Nobakht A et al., 2018). The separate scores for anxiety were not reported in Sabet et al. (Hemmati Sabet et al., 2013). Nowbi et al. used the Hospital Anxiety Depression Scale (HADS), and only the composite score 38.29 (SD 1.33) to 12.57 (SD 1.69) was reported (Nwobi et al., 2018). Hass et al. (2023) used the Shoan symptoms scale (SSQ) for anxiety and only reported scores at baseline, SSQ-14: 10.1 (SD 1.1) in the intervention and the control group of 272 patients showed at baseline, SSQ-14: 9.9 (SD 1.1) and did not mention follow-up mean and SD values (Haas et al., 2023). However, of the four studies focusing on anxiety, only one provided complete outcome data (Nobakht et al., 2018) (Table 2).

### Intervention design and delivery

CBT-based interventions across the 12 studies included various therapeutic components. In standard CBT, core techniques such as psychoeducation, cognitive restructuring, behavioral activation, and problem-solving were consistently used. In contrast, mixed CBT interventions integrated additional components, namely stress management and relaxation training, alongside cognitive restructuring and problem-solving, thereby extending the scope of standard CBT approaches.

The number of sessions received was similar across studies, with group-based therapies ranging from 6 to 11 sessions, and individual therapies spanning 6 to 12. Session duration was more varied, with group therapy typically lasting 1.5 to 2.5 h (Nwobi et al., 2018; Jalali et al., 2019; Pujiati and Gasem, 2022). In contrast, individual therapy sessions varied from 15 to 50 min (Chibanda et al., 2015; Chattopadhyay et al., 2017; Guo et al., 2020).

Therapies were delivered by healthcare professionals in four studies, including psychologists, experts in CBT, schema-certified therapists, and trained counselors. In the other four studies, therapy was provided by lay health workers and nurses. Innovative methods, such as web-based apps, were also utilized by a single study (Guo et al., 2020). However, three studies did not specify the therapy provider (Table 3).

**Table 3.** Therapy components Table 3. long description.

Author	Intervention name	Type	Provider	Duration	No of sessions	Setting	Components
Abbas et al. (2023)	CBT	Individual therapy	NR	NR	6	ART Clinic	Enhancing motivation, and reducing stigma through psychoeducation, cog- native restructuring, skill training, stress management, and relapse prevention
Chattopadhyay et al. (2017)	CBT	Group therapy	NR	30 min	8	Tertiary-care hospital of Kolkata	Module 1 – adherence training, consisting of two sessions, module 2 – behavioral activation (one session), module 3 – cognitive reconstruction (three sessions), module 4 – problem-solving (one session), and module 5 – relaxation training (one session)
Nobakht A et al. (2018)	CBT	Group therapy	Researcher, who was a student in MS counseling midwifery trained in the field of CBT.	NR	6	NR	NR
Safran et al. (2021)	CBT-AD	Individual therapy	Task-shared, nurse-delivered	NR	8	Public Clinics	(1) Life-steps adherence counseling; (2) introduction to CBT, psychoeducation about the nature of depression and motivational interviewing; (3) behavioral activation; (4) problem-solving; (5) relaxation training (≈ one session); and (6) review and relapse prevention
Hemmati Sabet et al. (2013)	CBT & stress management	Group therapy	NR	NR	NR	NR	NR
Nwobi et al. (2018)	Cognitive-behavioral stress management (MBSM)	Group therapy	Therapist	2.5 h.	10		Combined relaxation and imagery, progressive muscle relaxation, and meditation. Stress management, cognitive restructuring, coping strategies, and establishing a strong social network
Guo et al. (2020)	Cognitive-behavior stress management (MBSM)	Individual therapy	WeChat Web App	15 min	12	Online	Stress reduction management and coping skills such as muscle relaxation, breathing, and meditation
Chibanda et al. (2014)	CBT-based problem-solving therapy	Group therapy	6 trained peer counselors,	60 min	6	Private antenatal clinic	NR
Kaaya et al. (2022)	CBT & Problem-solving healthy options	Group therapy	Trained community-based lay facilitators		8		Problem-solving therapy (PST) during pregnancy and cognitive-behavioral therapy (CBT) after delivery
Haas et al. (2023)	Friendship bench-based problem-solving	Individual therapy	Lay health workers	NR	6 weeklies	Easily accessible settings such as health facilities, park benches, or clinic consultation rooms	Problem-solving, positive thinking
Pujiati et al. (2023)	Mindfulness-based cognitive-behavior therapy (MCBCT)	Group therapy	Trained enumerators with a background in nursing and mindfulness training	2 h.	8	Hospital	Stress reduction, based on a modified spiritual mindfulness-based cognitive therapy technique
Jalili et al. (2018)	Cognitive schema focused	Group therapy	Certified schema therapist	90 min	11	Positive clubs	Psychoeducation, three sessions based on cognitive technique testing the validity of schemas, reframing of schema confirmation evidence, listing pros and cons of patient’s coping strategies, conducting dialog between the “schema side” and the “Healthy side,” four sessions recognizing schema-triggering event

Studies used a diverse range of therapeutic approaches, with Group CBT used in five studies, while Individual CBT was implemented in two. Cognitive-Behavioral Stress Management (CBSM) and Problem-Solving Therapy interventions were applied in both group and individual formats. Other notable interventions included mindfulness-based cognitive therapy and cognitive schema-focused therapy (Table 3).

### Risk-of-bias assessment

Five studies were classified as “high risk,” three as “low risk,” and four as “uncertain or unclear risk.” Studies were further categorized based on quality criteria, which demonstrated low risk of bias relating to reporting, selection, and attrition. All RCTs noted the blinding of outcome assessors, although participant and provider blinding were generally absent. Supplementary Figures 1 and 2 show that there was a relatively low risk of reporting and attrition bias.

### Robins-I

Both included non-randomized controlled trials by Sabet (2012) and Jalali (2018) were judged to be at serious risk of bias overall, primarily due to confounding and participant selection bias arising from the absence of randomization and limited adjustment for baseline prognostic factors. Other domains, including outcome measurement and deviations from intended interventions, were judged to be at moderate risk (Supplementary Figure 3).

### Quality assessment of interventions using TIDIeR checklist

The quality of intervention reporting varied across studies, with some providing comprehensive details on intervention delivery and fidelity while others lacked transparency in critical domains (Supplementary Table 2).

### Publication bias

The funnel plot for the initial analysis (Supplementary Figure 3) shows asymmetry, with a clustering of studies with positive effect sizes, indicating potential publication bias. The underrepresentation of smaller studies with negative findings suggests selective reporting or non-publication. The clustering of studies outside the pseudo 95% confidence limits (dashed lines) further support the possibility of bias or heterogeneity.

### Meta-analysis: Mean (SD) depression scores

**Figure 2.**
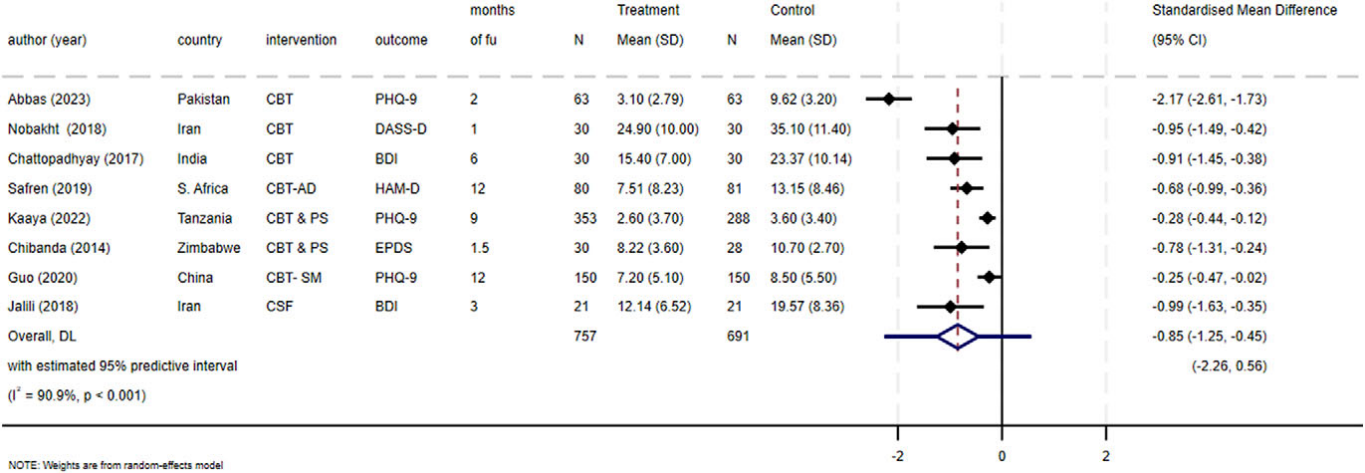
Overall, Forest Plot of pooled mean (SD) depression outcomes measure scores. Figure 2. long description.

Eight papers were included in the meta-analysis (Safren et al., 2009; Chibanda et al., 2014; Chattopadhyay et al., 2017; Nobakht et al., 2018; Jalali et al., 2019; Guo et al., 2020; Kaaya et al., 2022; Abbas et al., 2023). The overall meta-analysis revealed a pooled effect size of −0.85 (95% CI: −1.25, −0.45). This pooled effect size shows a statistically significant and large effect of CBT in reducing depressive symptoms among PLHIV in LMICs. The included studies demonstrated high heterogeneity (I^2^ = 93%, *p* < 0.001) (Figure 2).

### Subgroup analysis of intervention design characteristics

The subgroups analysis revealed that standard CBT demonstrated a larger effect size of −1.18 (95% CI: −1.68 to −0.47) in comparison with the mixed CBT-based approach, which showed a moderate effect size of −0.42 (95% CI: −0.67 to −0.17) (Supplementary Figure 4). Interventions delivered by mental health providers versus non-mental health providers also showed a large, pooled effect size compared to moderate effect size, respectively (−1.18 (95% CI: −1.75 to −0.60) vs. −0.37 (−0.58, −0.15)). When the providers were categorized by those with or without a background in mental health, the mental health providers’ group effect size, compared with the non-mental health providers, showed a slight difference from the effect size of −0.37 (−0.58, −0.15) (Supplementary Figure 5).

Interventions comprising six sessions yielded a significant pooled effect size of −1.31 (95% CI: −2.22 to −0.40). In contrast, interventions with more than six sessions showed a moderate statistically significant effect size of −0.52 (95% CI: −0.77 to −0.26) (Supplementary Figure 6). Subgroup analyses based on follow-up duration indicated that short-term follow-up (<6 months) was associated with a larger pooled effect size (*g* = −1.18, 95% CI: −1.75 to −0.60). In contrast, studies with long-term follow-up periods between (≥6 months to 12 months) demonstrated a smaller, though still statistically significant, pooled effect size (*g* = −0.37, 95% CI: −0.58 to −0.15). To account for potential heterogeneity arising from different follow-up durations, we explored the impact of follow-up timing through subgroup and/or sensitivity analyses (where applicable), which showed that the overall conclusions were robust and not driven by a specific time point. These findings suggest the magnitude of any effect may be difficult to sustain over the long term (Supplementary Figure 7). Subgroup analysis by delivery format showed that individual CBT had a larger pooled effect size (SMD = −1.01, 95% CI: −2.00 to −0.03) compared with group CBT (SMD = −0.73, 95% CI: −1.11 to −0.35). Both formats were associated with significant reductions in depressive symptoms, with the effect of individual CBT suggesting some great benefit (Supplementary Figure 8).

## Discussion

This systematic review and meta-analysis provide evidence supporting the effectiveness of CBT interventions in reducing depressive symptoms among PLHIV in low- and middle-income countries (LMICs). Across all included studies, intervention groups consistently outperformed control conditions in reducing depressive symptoms and, where reported, improving treatment adherence. Regarding the specific characteristics of effective interventions, our findings suggest that greater benefits may be observed with standard CBT delivered by trained mental health professionals or CBT specialists, and that interventions comprising approximately six sessions may be optimal. The mode of delivery, individual versus group, did not appear to substantially influence effectiveness, indicating flexibility in delivery formats without compromising outcomes. These findings suggest that while specialist-delivered, well-structured CBT may yield stronger effects, adapted delivery models may still be viable in resource-constrained LMIC settings.

The implementation of CBT in LMICs occurs within health systems that differ markedly from those in HICs, particularly with respect to workforce shortages, limited access to specialist mental health providers, high patient loads, and competing clinical priorities within HIV care. In many LMICs, mental health services are under-resourced, and depression among PLHIV is often underdiagnosed and undertreated (Patel et al., 2018; WHO, 2018). These structural constraints necessitate adaptations such as brief interventions, task-shifting to non-specialist providers, and integration of mental health care within existing HIV services, rather than reliance on specialist-only delivery models commonly used in HICs.

The integration of mental health interventions, particularly CBT, into HIV care platforms in LMICs represents a promising and pragmatic approach to addressing the mental health burden among PLHIV. Evidence from Safren et al. (2021), conducted in primary care HIV services in Khayelitsha, South Africa, demonstrates that CBT-based interventions can be effectively embedded within routine HIV services, leading to significant improvements in depression outcomes. This aligns with WHO mhGAP recommendations and broader global mental health literature advocating for integrated, task-shared care models in LMICs (WHO, 2017; Singla et al., 2017; Patel et al., 2018).

Our subgroup analyses revealed that standard CBT demonstrated larger effect sizes compared to CBT-based or adapted interventions. This finding mirrors evidence from HIC-based meta-analyses, including Cuijpers et al. (2010), which reported an effect size of 0.69 for CBT relative to other psychological therapies in more affluent countries. Similarly, provider expertise was associated with stronger effects, with interventions delivered by trained psychologists or CBT specialists outperforming those delivered by lay health workers or nurses, again consistent with HIC literature. In contrast, LMIC contexts often necessitate task-shifting approaches, where non-specialists deliver psychological interventions under supervision. While these approaches may yield smaller effect sizes, they remain essential for scalability and equity in LMICs and should be interpreted within the context of feasibility rather than direct equivalence with specialist-led HIC models (Singla et al., 2017; Patel et al., 2018).

Although point estimates suggested that individual CBT may be superior to group CBT, wide confidence intervals indicate imprecision, likely due to a limited number of contributing studies and potential outliers (e.g., Abbas et al., 2023). Overall, individual versus group CBT did not significantly alter outcomes. This aligns with findings from HIC studies (Cuijpers et al., 2010; Krishna et al., 2015; Karyotaki et al., 2017), but diverges from findings in sub-Saharan Africa, where Nakimuli-Mpungu et al. (2021) reported moderate effects of group-based support interventions among PLHIV. This divergence highlights the importance of contextual and cultural factors in LMICs, where group-based interventions may offer additional benefits such as peer support, stigma reduction, and cost efficiency that are less prominent in HIC settings.

The finding that brief CBT interventions were effective supports the practicality of short, focused treatments in low-resource settings. Consistent with HIC evidence (Cuijpers et al., 2013a; Cuijpers et al., 2023), session number was not strongly associated with outcomes once covariates were controlled for. However, from an LMIC perspective, shorter interventions may enhance feasibility, reduce attrition, and improve scalability within overburdened HIV care systems.

Follow-up duration moderated intervention effects, with larger benefits observed at shorter follow-up periods (<6 months) and diminishing effects beyond 12 months. This pattern is consistent with HIC-based meta-analyses (Karyotaki et al., 2017; Keles & Idsoe, 2018; Chan et al., 2025) but poses particular challenges in LMICs, where long-term mental health follow-up is often limited. Scalable strategies such as booster sessions, group maintenance sessions, or brief digital follow-ups may offer feasible approaches to sustaining gains in LMIC settings.

### Anxiety outcomes and evidence gaps in LMICs

Only four studies assessed anxiety outcomes, and these were limited by small numbers, heterogeneous measures, and insufficient reporting to permit meta-analysis. Anxiety was often reported alongside depression or within composite scales such as HADS or DASS-21, limiting interpretability. Overall, there remains a paucity of evidence on the effectiveness of CBT for anxiety among PLHIV in LMICs, consistent with previous reviews highlighting substantial gaps in anxiety-focused intervention research in these settings (Spies et al., 2013; van Luenen et al., 2018).

This review has several strengths. By exclusively focusing on controlled trials in LMICs the review ensures a high level of methodological rigor and reliability of findings in this specific context. Additionally, the targeted focus on CBT for depression and anxiety allows for a more precise evaluation of this specific intervention, enhancing the clinical applicability and relevance for mental health practitioners working with populations in LMICs. However, we acknowledge several limitations in this work. First, the high heterogeneity indicates the variability across various psychological scales for depression outcomes in the included studies, even though all focused on CBT in some form. Second, depression remains the focus of research in this area, and data on the role of CBT on anxiety in PLHIV in LMIC remain limited. To address this, future studies should incorporate broader mental health assessments to capture a more comprehensive picture of intervention effects. Third, the presence of publication bias suggests that studies with negative or null results may be underrepresented in this field. Finally, the quality of the studies included varied, with some studies lacking clear reporting on key methodological aspects, such as small sample size, blinding, and allocation concealment. This was accounted for by conducting subgroup analyses and using risk-of-bias tools to assess the methodological rigor of each study, thereby ensuring that the main findings were not unduly influenced by lower-quality evidence but to use data with caution. Lastly, the absence of a second reviewer at the full-text screening represents a limitation of our review.

In conclusion, our findings reinforce the effectiveness of standard CBT interventions in improving depression outcomes in PLHIV in LMIC. It underscores the importance of incorporating CBT into routine care for PLHIV experiencing depression in LMIC settings. Tailoring interventions based on session structure, provider qualifications, and therapy intensity may enhance effectiveness and improve overall mental health and quality of life for this vulnerable population. Though more evidence is required to determine if a similar benefit can be seen in those experiencing anxiety, the role in depression would suggest it is likely. Where resources are allowed, CBT should be implemented in PLHIV in LMICs and the design of such treatment tailored based on our findings, where culturally appropriate.

## Supporting information

10.1017/gmh.2026.10223.sm001 Mughal Azeemi et al. supplementary material 1 Mughal Azeemi et al. supplementary material

10.1017/gmh.2026.10223.sm002 Mughal Azeemi et al. supplementary material 2 Mughal Azeemi et al. supplementary material

## Data Availability

All data analyzed in this study were obtained from published studies cited in the manuscript and available within article references, or their supplementary material.

## Supplementary material

The supplementary material for this article can be found at http://doi.org/10.1017/gmh.2026.10223.

## Figure 1. Long description

At the top, a yellow header states Identification of studies via databases and registers. The main vertical path begins with Records identified through databases MEDLINE n equals 375, E M B A S E n equals 1233, Psyc I N F O n equals 389, C I N H A L n equals 290, Cochrane n equals 341. An arrow leads down to Records screened and imported to Rayyan n equals 929. A rightward arrow points to Records excluded as irrelevant n equals 769, listing reasons: Different intervention n equals 108, Different outcomes n equals 83, Different countries n equals 289, Wrong publication type n equals 269. The main path continues down to Records assessed for eligibility n equals 237. Another rightward arrow points to Full-text articles excluded, with reasons n equals 145: Different outcomes n equals 15, Wrong publication types n equals 20, Different countries n equals 82, American n equals 20, Wrong population n equals 11. The central path continues to Full-text articles assessed for eligibility n equals 160, then to Studies included in the review n equals 12. A rightward arrow points to Excluded studies before M A n equals 4, with reasons: Absence of Mean and S D n equals 2, Statistical inappropriate outcome n equals 1, Measurement not specifically provided D and A scores n equals 1. The final box at the bottom is Studies included in the Meta-analysis n equals 8. The left margin labels each stage vertically: Identification, Screening, Eligibility, Included.

Navigate back to Figure 1..

## Table 1. Long description

Beginning at the top row, column headers are Author and year, Country, N, Study design, Age, Male percentage, HIV diagnosis, Depression or Anxiety (D and A), Intervention and number in intervention (NI), and Control group. Each subsequent row details individual studies: Abbas et al. 2023 from Pakistan with 126 participants, RCT design, age range 20–55, mean 37.5 (10.11), 41 percent male, registered at ART clinic, PHQ-9 for depression, B-CBT intervention with 63, wait-list control with 63. Chattopadhyay et al. 2017 from India, 60 participants, RCT, age range 20–60, mean 40 (11.55), 65 percent male, HIV cases at referral center, BDI for depression, CBT intervention with 30, TAU control with 30. Nobakht A et al. 2018 from Iran, 288 participants, RCT, mean age 30 (5), 100 percent female, HIV-positive women, DASS-21 for depression, CBT intervention with 14, TAU control with 14. Safren et al. 2021 from South Africa, 163 participants, RCT, age not reported, 29.8 percent male, HIV RNA viral load measured, MINI for depression, CBT-AD intervention with 80, enhanced TAU control with 81. Hemmati Sabet et al. 2013 from Iran, 42 participants, semi-experimental pre-post design, age not reported, 100 percent male, diagnosed HIV-positive men, D and A and stress scale, CBT intervention with 21, stress management control with 21. Nwobi et al. 2018 from Nigeria, 28 participants, RCT, mean age I equals 45.71 plus or minus 3.31, C equals 43.57 plus or minus 3.63, I equals 5 males (41.7 percent), C equals 7 males (58.3 percent), diagnosed HIV patients, HADS for depression, CBSM intervention with 14, no treatment control with 14. Guo et al. 2020 from Guangzhou, China, 300 participants, RCT, age at least 18 years, mean 28 (5.8), 92 percent male, HIV seropositive, CES-D scale score at least 16, CBSM intervention, WeChat platform control. Chibanda 2014 from Zimbabwe, 58 participants, RCT, mean age 25, 0 percent male, HIV diagnosis not reported, DSM-IV for depression, PST intervention with 30, TAU and amitriptyline control with 28. Kaaya et al. 2022 from Tanzania, 742 participants, cluster RCT, mean age I equals 29.8 (5.5), C equals 29.5 (5.3), 0 percent male, HIV-positive pregnant women, PHQ-9 for depression, healthy options including PST and CBT plus EUDC intervention with 395, EUDC mhGap guidelines control with 347. Haas et al. 2023 from Zimbabwe, 516 participants, open-label pragmatic cluster RCT, mean age 45.6, 15.1 percent male, diagnosed HIV at ART clinics, SSQ-14 and PHQ-9 for depression, Friendship Bench intervention with 244 across 8 clusters, ESC intervention control with 272 across 8 clusters. Pujiati et al. 2022 from Indonesia, 62 participants, RCT, mean age I equals 35.10 plus or minus 6.01, C equals 33.35 plus or minus 7.06, 54.2 percent male, PLHIV on ART, psychiatrist diagnosis for depression, MBCT plus hypnosis intervention with 31, MBCT control with 31. Jalali et al. 2019 from Iran, 42 participants, between-groups pre-posttest, mean age I equals 32.60 plus or minus 4.95, C equals 30.96 plus or minus 3.35, 100 percent male, male prisoners with HIV, clinical interviews and BDI score greater than 14, CSF intervention with 21, WLC group control with 21. Abbreviations are defined in the table footnote, including BDI as Beck Depression Inventory, CBSM as Cognitive-Behavioral Stress Management, MBCT as Mindfulness-Based Cognitive Therapy, PST as Problem-solving therapy, CES-D as Center for Epidemiologic Studies-Depression Scale, DASS-21 as Depression Anxiety and Stress Scales, DSM-IV as Diagnostic and Statistical Manual of Mental Disorders Fourth Edition, HADS as Hospital Anxiety and Depression Scale, PLHIV as people with human immunodeficiency virus, SSQ as Shona Symptoms Questionnaire, PHQ-9 as Patient Health Questionnaire-9, TAU as treatment as usual, WLC as wait-list control, ESC as enhanced standard care, EUDC as enhanced usual care for depression.

Navigate back to Table 1..

## Table 2. Long description

The table lists studies by lead author and year, country, and sample size. For each study, outcome measure, follow-up length in months, and number added in analysis are shown. Intervention group columns include baseline mean with standard deviation and post mean with standard deviation. Control group columns include sample size, baseline mean with standard deviation, and post mean with standard deviation. For example, Abbas et al. 2023 from Pakistan used PHQ-9, with 63 participants, 2 months follow-up, 80 analyzed, intervention baseline mean 11.70 (4.23), post mean 3.10 (2.79); control baseline mean 9.92 (3.42), post mean 9.62 (3.20). Chattopadhyay et al. 2017 from India used BDI and adherence, with 30 participants, 6 months follow-up, intervention baseline mean 20.53 (9.71), post mean 15.40 (7.00); control baseline mean 21.33 (9.45), post mean 23.37 (10.14). Nobakht A et al. 2018 from Iran used DASS-D, 30 participants, 1 month, intervention baseline mean 31.1 ± 11.9, post mean 24.9 ± 10; control baseline mean 36.1 ± 12.1, post mean 35.1 ± 11.4. Safren et al. 2021 from South Africa used HAM-D, 80 participants, 12 months, intervention baseline mean 23.75 (0.83), post mean 7.51 (0.92); control baseline mean 25.76 (0.83), post mean 13.15 (0.94). Hemmati Sabet et al. 2013 from Iran used DASS-D, 16 participants, intervention baseline mean 29.07 (6.181), post mean 23.73 (4.949); control baseline mean 6.038 (27.4), post mean 4.926 (28). Nwobi et al. 2018 from Nigeria used HADS, 14 participants, 3 months, intervention baseline mean 38.29 (1.33), post mean 12.57 (1.69); control baseline mean 37.86 (1.23), post mean 38.36 (1.22). Guo et al. 2020 from China used CES-D and PHQ-9, 150 participants, 12 months, intervention baseline mean 23.9 (6.4), post mean 18.1 (11.3); control baseline mean 24.3 (6.9), post mean 23.3 (12.4). Chibanda et al. 20114 from Zimbabwe used EPDS, 30 participants, 1.5 months, intervention baseline mean 17.3 + 3.7, post mean 8.22 + 3.6; control baseline mean 17.9 + 3.9, post mean 10.7 + 2.7. Kaaya et al. 2022 from Tanzania used PHQ-9, 353 participants, 9 months, intervention baseline mean 11.3 (3.2), post mean 2.6 (3.7); control baseline mean 11.6 (3), post mean 3.6 (3.4). Haas et al. 2023 from Zimbabwe used PHQ-9, 244 participants, 12 months, intervention baseline mean 8.3 (3.6), post mean NA; control baseline mean 7.2 (3.2), post mean NA. Pujiati et al. 2022 from Indonesia, 31 participants, intervention baseline mean 35.0 ± 5.1, post mean 22.9 ± 5.4; control baseline mean 34.3 ± 4.8, post mean 29.3 ± 4.9. Jalili et al. 2019 from Iran used BDI, 21 participants, intervention baseline mean 25.04 (7.22), post mean 12.14 (6.52); control baseline mean 18.14 (9.32), post mean 19.57 (8.36). Missing data and not reported values are indicated as NA or NR.

Navigate back to Table 2..

## Table 3. Long description

Beginning at the top row, the columns are Author, Intervention name, Type, Provider, Duration, Number of sessions, Setting, and Components. The first row lists Abbas et al. 2023, CBT, individual therapy, NR, NR, 6 sessions, ART Clinic, with components including enhancing motivation and reducing stigma through psychoeducation, cognitive restructuring, skill training, stress management, and relapse prevention. The second row, Chattopadhyay et al. 2017, CBT, group therapy, NR, 30 minutes, 8 sessions, tertiary-care hospital of Kolkata, components are module 1 adherence training (two sessions), module 2 behavioral activation (one session), module 3 cognitive reconstruction (three sessions), module 4 problem-solving (one session), module 5 relaxation training (one session). The third row, Nobakht A et al. 2018, CBT, group therapy, researcher trained in CBT, NR, 6 sessions, NR, NR. The fourth row, Safran et al. 2021, CBT-AD, individual therapy, task-shared nurse-delivered, NR, 8 sessions, public clinics, components are life-steps adherence counseling, introduction to CBT, psychoeducation about depression and motivational interviewing, behavioral activation, problem-solving, relaxation training (approximately one session), review and relapse prevention. The fifth row, Hemmati Sabet et al. 2013, CBT and stress management, group therapy, NR, NR, NR, NR, NR. The sixth row, Nwobi et al. 2018, cognitive-behavioral stress management (MBSM), group therapy, therapist, 2.5 hours, 10 sessions, blank setting, components are combined relaxation and imagery, progressive muscle relaxation, meditation, stress management, cognitive restructuring, coping strategies, and establishing a strong social network. The seventh row, Guo et al. 2020, cognitive-behavior stress management (MBSM), individual therapy, WeChat Web App, 15 minutes, 12 sessions, online, components are stress reduction management and coping skills such as muscle relaxation, breathing, and meditation. The eighth row, Chibanda et al. 2014, CBT-based problem-solving therapy, group therapy, 6 trained peer counselors, 60 minutes, 6 sessions, private antenatal clinic, NR. The ninth row, Kaaya et al. 2022, CBT and problem-solving healthy options, group therapy, trained community-based lay facilitators, blank duration, 8 sessions, blank setting, components are problem-solving therapy during pregnancy and CBT after delivery. The tenth row, Haas et al. 2023, friendship bench-based problem-solving, individual therapy, lay health workers, NR, 6 weekly sessions, settings include health facilities, park benches, or clinic consultation rooms, components are problem-solving and positive thinking. The eleventh row, Pujiati et al. 2022, mindfulness-based cognitive-behavior therapy (MCBCT), group therapy, trained enumerators with nursing and mindfulness training, 2 hours, 8 sessions, hospital, components are stress reduction based on a modified spiritual mindfulness-based cognitive therapy technique. The twelfth row, Jalili et al. 2018, cognitive schema focused, group therapy, certified schema therapist, 90 minutes, 11 sessions, positive clubs, components are psychoeducation, three sessions based on cognitive technique testing the validity of schemas, reframing of schema confirmation evidence, listing pros and cons of patient’s coping strategies, conducting dialog between the schema side and the healthy side, four sessions recognizing schema-triggering event.

Navigate back to Table 3..

## Figure 2. Long description

From left to right, the table lists author and year, country, intervention, outcome, months of follow-up, sample size, and mean with standard deviation for both treatment and control groups. The rightmost section displays a forest plot of standardized mean differences with 95 percent confidence intervals for each study. Study rows are: Abbas 2023 (Pakistan, C B T, P H Q dash 9), Nobakht 2018 (Iran, C B T, D A S S dash D), Chattopadhyay 2017 (India, C B T, B D I), Safren 2019 (South Africa, C B T dash A D, H A M dash D), Kaaya 2022 (Tanzania, C B T and P S, P H Q dash 9), Chibanda 2014 (Zimbabwe, C B T and P S, E P D S), Guo 2020 (China, C B T dash S M, P H Q dash 9), Jalili 2018 (Iran, C S F, B D I). Sample sizes range from 21 to 150 per group. Treatment means are consistently lower than control means. The forest plot shows all studies with negative standardized mean differences, favoring treatment, ranging from minus 2.17 to minus 0.25, except for one study with a confidence interval crossing zero. The overall effect is minus 0.85 with a 95 percent confidence interval from minus 1.25 to minus 0.46. The diamond at the bottom represents the pooled effect size. The note below states weights are from a random effects model, with I squared equal to 90.9 percent and p less than 0.001.

Navigate back to Figure 2..
