# Supplementary material for: The effectiveness of cognitive behavioral therapy-based interventions for depression and anxiety in people living with HIV in low- and middle-income countries: A systematic review and meta-analysis
Source: Glob Ment Health (Camb). 2026 May 13;13:e124. doi: 10.1017/gmh.2026.10223 (PMC13312370; doi:10.1017/gmh.2026.10223)
Supplement: Mughal Azeemi et al. supplementary material 1 — Mughal Azeemi et al. supplementary material [file S2054425126102234sup001.docx]

**
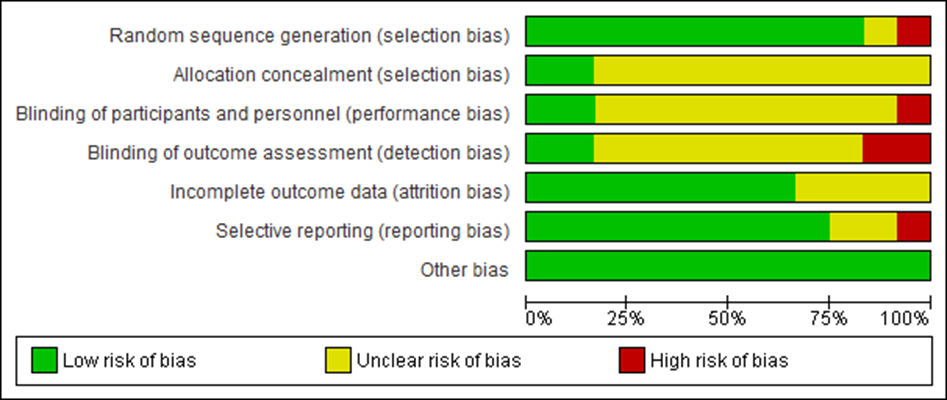
**

Supplementary Figure 1: Risk of Bias Graph

**
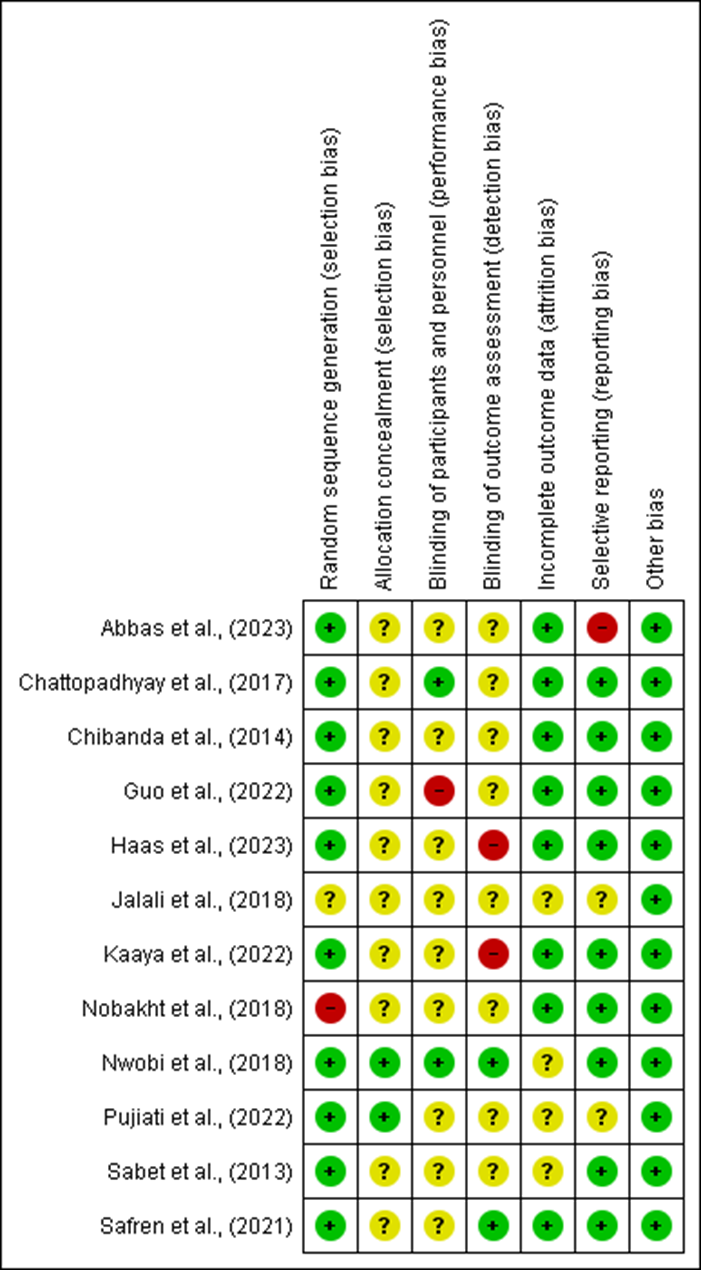
**

Supplementary Figure 2: Risk of Bias (ROB) summary


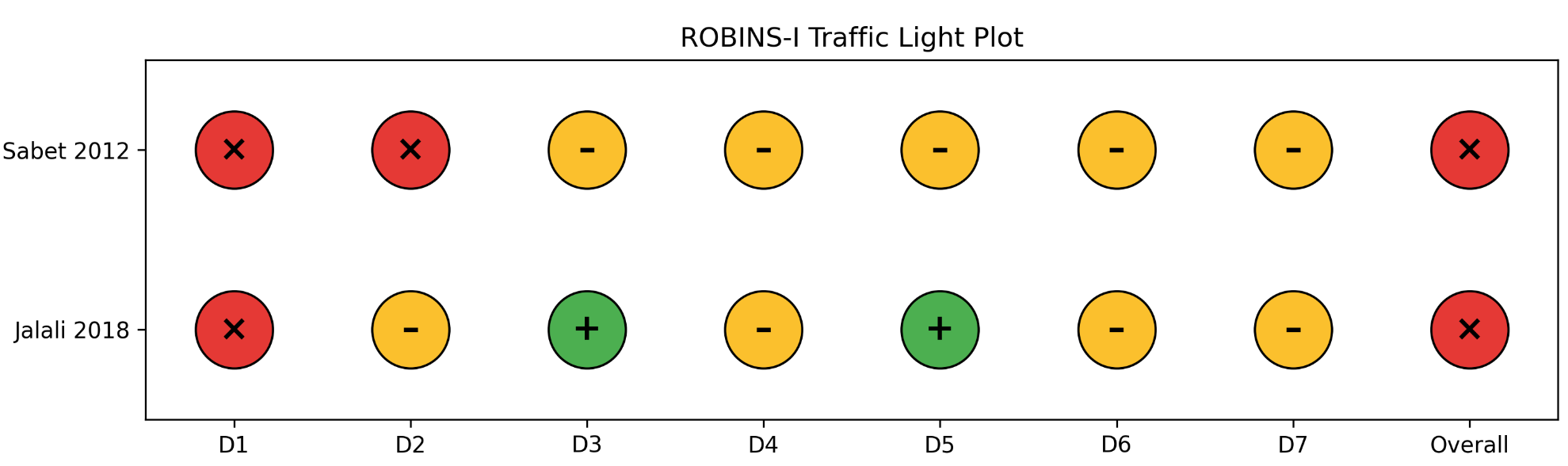


**Supplementary Figure 3: Risk of bias of a non-randomised controlled trial**

**Columns (D1–D7):**

- **D1:** Bias due to confounding
- **D2:** Bias in selection of participants
- **D3:** Bias in classification of interventions
- **D4:** Bias due to deviations from intended interventions
- **D5:** Bias due to missing data
- **D6:** Bias in measurement of outcomes
- **D7:** Bias in selection of the reported result

**
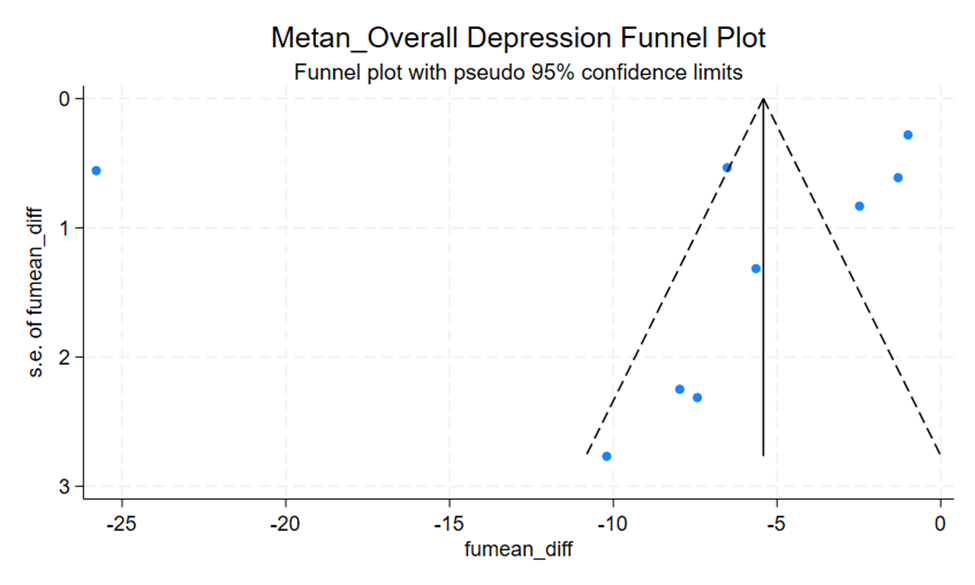
**

Supplementary Figure 4: Funnel plot publication bias for the overall depression outcome

Supplementary Figure 5: Subgroup analysis of intervention design characteristics (CBT vs Mixed CBT-based approach)

Supplementary Figure 6: Subgroup analysis of Providers' mental health vs non-mental health

Supplementary Figure 7: Subgroup analysis on number of sessions (0-6 vs >6)

Supplementary Figure 8: Subgroup analysis on follow-up durations (short-term <6 months vs long-term >6 to 12 months)

Supplementary Figure 9: Subgroup analysis of individual vs group CBT
