# Supplementary material for: The effectiveness of cognitive behavioral therapy-based interventions for depression and anxiety in people living with HIV in low- and middle-income countries: A systematic review and meta-analysis
Source: Glob Ment Health (Camb). 2026 May 13;13:e124. doi: 10.1017/gmh.2026.10223 (PMC13312370; doi:10.1017/gmh.2026.10223)
Supplement: Mughal Azeemi et al. supplementary material 2 — Mughal Azeemi et al. supplementary material [file S2054425126102234sup002.docx]

| **#** | **Query** |
| --- | --- |
| S1 | TI HIV1 OR AB HIV1 OR SU HIV1 |
| S2 | TI HIV2 OR AB HIV2 OR SU HIV2 |
| S3 | TI HIV OR AB HIV OR SU HIV |
| S4 | TI "acquired immunodeficiency" W0 syndrome* OR AB "acquired immunodeficiency" W0 syndrome* OR SU "acquired immunodeficiency" W0 syndrome* |
| S5 | TI "acquired immune deficiency" W0 syndrome* OR AB "acquired immune deficiency" W0 syndrome* OR SU "acquired immune deficiency" W0 syndrome* |
| S6 | TI HIV/AIDS OR AB HIV/AIDS OR SU HIV/AIDS |
| S7 | TI ( (AIDS N2 (associate* or relate*) ) OR AB ( (AIDS N2 (associate* or relate*) ) OR SU ( (AIDS N2 (associate* or relate*) ) |
| S8 | (MH "HIV+") |
| S9 | S1 OR S2 OR S3 OR S4 OR S5 OR S6 OR S7 OR S8 |
| S10 | (MH "Cognitive Behavioral Therapy+") |
| S11 | TI (Cogniti*) N2 (therap* or rehab* or interven* or program* ) OR AB ( (Cogniti*) N2 (therap* or rehab* or interven* or program* ) OR SU ( (Cogniti*) N2 (therap* or rehab* or interven* or program*) |
| S12 | TI CBT OR AB CBT OR SU CBT |
| S13 | TI cognitive W0 behavio* OR AB cognitive W0 behavio* OR SU cognitive W0 behavio* |
| S14 | S10 OR S11 OR S12 OR S13 |
| S15 | (MH "Depressive Disorder+") |
| S16 | (MH "Anxiety Disorders+") |
| S17 | (MH "Phobia, Social") |
| S18 | TI Depress* OR AB Depress* OR SU Depress* |
| S19 | TI Anxi* OR AB Anxi* OR SU Anxi* |
| S20 | TI Fear* OR AB Fear* OR SU Fear* |
| S21 | TI Stress* OR AB Stress* OR SU Stress* |
| S22 | TI Distress* OR AB Disstress* OR SU Distress* |
| S23 | TI Phobia* OR AB Phobia* OR SU Phobia* |
| S24 | TI Common mental" W0 disorder* OR AB Common mental" W0 disorder* OR SU Common mental" W0 disorder* |
| S25 | TI CMD* OR AB CMD* OR SU CMD* |
| S26 | S15 OR S16 OR S17 OR S18 OR S19 OR S20 OR S21 OR S22 OR S23 OR S24 OR S25 |
| S27 | S9 AND S14 AND S26 |

**Supplementary** **Table 1: Systematic Review Search Strings**

**Medline Search Strategy**

In the search strategy, I combined and used AND – OR

| **TIDIER Item** | **Description** | **Findings from Included Studies** |
| --- | --- | --- |
| **1. Brief Name** | Cognitive Behavioral Therapy (CBT) for depression and anxiety among PLHIV. | **CBT was explicitly identified** as the primary intervention in **5 out of 13 studies (38%)**, while **8 studies (62%)** implemented CBT-based interventions (e.g., CBT-based stress management, CBT-problem solving, and cognitive therapy-based mindfulness). and provided a **clear theoretical justification** for their selection over other psychological treatments. |
| **2. Why (Rationale, Theory, or Goal of the Intervention)** | CBT aims to modify maladaptive thoughts and behaviors to reduce depression and anxiety. PLHIV experience unique stressors that CBT may address effectively. | These studies cited CBT’s **evidence-based effectiveness** in addressing depression, anxiety, and psychosocial challenges in PLHIV. |
| **3. What (Materials Used in the Intervention)** | - CBT therapy manuals and workbooks.  - Therapist guides, worksheets, and digital tools.  - Psychoeducational materials addressing HIV-related stigma and coping. | **Six studies (46%)** explicitly described intervention materials, including **manuals, worksheets, psychoeducational resources, pamphlets, or brochures**. **One study (7%)** provided an access link to a manual. **7 studies (54%) did not report on specific materials**. Additionally, **one study (7%) implemented a digital/web-based CBT intervention** but lacked detailed descriptions of online tools or app-based platforms, limiting replicability. |
| **4. Who Provided (Intervention Deliverers)** | - Licensed mental health professionals (psychologists, psychiatrists, counselors, social workers).  - Trained lay counselors or peer support workers. | **Nine studies (69%)** reported the qualifications of intervention deliverers, including **doctoral-level candidates, clinical psychologists, CBT experts, counselors, and other healthcare providers**. However, **4 studies (31%) did not specify the provider**, raising concerns about fidelity. **5 studies (38%)** using **lay counselors** provided additional training protocol details. |
| **5. How (Delivery Mode)** | - Individual or group therapy sessions.  - Teletherapy (video or phone-based CBT).  - Self-guided CBT (mobile apps, web platforms). | CBT was delivered through: **Individual therapy (n = 3, 23%)**, **Group therapy (n = 9, 69%)**, and **Teletherapy (n = 1, 7%)**. While all studies described the **general session format**, **only 6 studies (46%)** provided detailed descriptions of **session structure, duration, and goals**. The **single teletherapy study (7%)** lacked information on engagement strategies and platform effectiveness. |
| **6. Where (Location or Setting of Intervention Delivery)** | - Hospital or clinic-based settings (HIV care centers, mental health clinics).  - Community-based programs.  - Online/telehealth interventions. | **Three studies (23%)** were conducted in **ART clinics**, **2 studies (15%)** in **tertiary care hospitals**, **4 studies (30%)** in **community-based settings**, and **1 study (8%)** used a **fully digital CBT model**. **Primary care urban clinics** were used in **1 study (7%)**. |
| **7. When and How Much (Intervention Dose and Schedule)** | - Duration: 6–12 weeks.  - Frequency: Weekly or biweekly sessions (60–90 minutes).  - Follow-up sessions for maintenance. | The intervention duration ranged from **6 to 12 weeks**: **6 weeks (n = 5, 38%)**, **8–12 weeks (n = 8, 62%)**. Studies with **shorter CBT durations (6 weeks) had higher symptom improvement and lower retention rates**, while **longer interventions (12 weeks) showed moderate symptom reduction but increased dropout rates**. Only **6 studies (46%)** provided a clear justification for the session length. |
| **8. Tailoring (Modifications for Specific Populations)** | - CBT adapted for HIV-related stigma, trauma, and treatment adherence.  - Culturally sensitive adaptations.  - Modified formats for individuals with cognitive impairment. | **Three studies (23%)** reported tailoring CBT interventions to address **HIV-related stigma, trauma, and adherence challenges**, improving engagement. **3 studies (23%)** explicitly designed their CBT intervention using **Beck's CBT principles**. However, **7 studies (54%)** did not mention any adaptations. |
| **9. Modifications (Changes to the Intervention Over Time)** | - Adjustments to delivery mode (e.g., teletherapy due to COVID-19).  - Integration of mindfulness techniques.  - Addition of peer-led support components. | **One study (7%)** reported modifying the intervention mid-study to increase **support intensity, coping strategies, and peer support**, incorporating **income-generating activities**. **5 studies (39%) did not modify the intervention**, and **7 studies (54%) did not specify whether modifications were made**, making it difficult to assess population-specific adjustments. |
| **10. How Well (Adherence and Fidelity to the Intervention)** | - Fidelity monitoring through session recordings, checklists, or supervision.  - Patient adherence through session attendance and homework completion. | **Six studies (46%)** used fidelity monitoring strategies: **Session recordings (n = 3, 23%)**, **Therapist adherence checklists (n = 4, 31%)**, and **Supervision/training assessments (n = 5, 38%)**. **7 studies (54%) lacked fidelity assessments**, raising concerns about the consistency of CBT implementation. |
| **11. How Well (Implementation and Follow-Up Assessment)** | - Follow up at 3, 6, 12 and 36 months.  - Standardized scales used (e.g., PHQ-9 for depression, GAD-7 for anxiety).  - Outcomes on quality of life, treatment adherence, and psychosocial factors. | Follow-up periods varied: **Short-term (6 months) (n = 5, 38%)**, **Mid-term (8–10 months) (n = ?)**, and **Long-term (12–36 months) (n = 1, 7%)**. Studies with **short-term follow-ups (6 months) reported sustained symptom reduction**, while **4 studies (31%) did not report follow-up outcomes**, making it difficult to assess long-term CBT effectiveness. |

**Supplementary Table 2: TIDIER Checklist for Systematic Review on CBT for Depression and Anxiety in PLHIV**
