# Supplementary material for: The effectiveness of cognitive behavioral therapy-based interventions for depression and anxiety in people living with HIV in low- and middle-income countries: A systematic review and meta-analysis
Source: Glob Ment Health (Camb). 2026 May 13;13:e124. doi: 10.1017/gmh.2026.10223 (PMC13312370; doi:10.1017/gmh.2026.10223)
Supplement: Peer review history

# Reviewer report: Review: The effectiveness of cognitive behavioral therapy-based interventions for depression and anxiety in people living with HIV in low- and middle-income countries: A systematic review and meta-analysis — R0/PR2

**DOI:** 10.1017/gmh.2026.10223.pr2

Dear Author

While thanking you for your valuable research, here are a few suggestions:

In lines 75 to 78, you did not use references in front of the sentences. It is suggested that you use the following related reference: Abdollahpour, Sedigheh, et al. “The efficacy of cognitive behavioural therapy on stress, anxiety and depression of infertile couples: a systematic review and meta-analysis.” Journal of Obstetrics and Gynaecology 42.2 (2022): 188-197.

Many other sentences in the introduction section are without references. For example, in line 103, you could use the following reference: Abdollahpour, Sedigheh, et al. “Effect of brief cognitive behavioral counseling and debriefing on the prevention of post-traumatic stress disorder in traumatic birth: a randomized clinical trial.” Community mental health journal 55.7 (2019): 1173-1178.

Abdollahpour, Sedigheh, et al. “The effect of debriefing and brief cognitive-behavioral therapy on postpartum depression in traumatic childbirth: a randomized clinical trial.” Journal of Midwifery & Reproductive Health 6.1 (2018).

In the search strategy, include the words you combined and used AND - OR

Did you not subgroup the results based on the number of counseling sessions? In the meta-analysis, were the depression questionnaires the same or different? How did you meta-analyze the results when the questionnaires were not the same?

Why did you not draw the risk of bias and forest plot?

Some of the references are old and need to be revised.

# Reviewer report: Review: The effectiveness of cognitive behavioral therapy-based interventions for depression and anxiety in people living with HIV in low- and middle-income countries: A systematic review and meta-analysis — R0/PR3

**DOI:** 10.1017/gmh.2026.10223.pr3

Thank you for the opportunity to review this systematic review and meta-analysis. The study addresses an important topic; however, substantial revisions are required before it can be considered for publication in this journal. Please find my comments below.

• In the introduction, the authors attempt to establish a rationale for conducting this review; however, this section could be strengthened by providing a clearer explanation of why findings from high-income countries (HICs) may not be directly transferable to low- and middle-income countries (LMICs) in this context.

• A clear definition of LMICs should be provided, including how this classification was determined.

• Line 61: There is a typographical error—“In Pakistan” should replace “n Pakistan.”

• Additional detail is required in the methods section regarding the screening and selection process. Specifically:

o Were any diagnostic criteria applied for HIV?

o Were specific questionnaires or classification systems required for the assessment of depression and/or anxiety?

o What was the rationale for including both randomized controlled trials (RCTs) and non-randomized controlled studies (non-RCTs)?

o How was LMIC status determined?

o If studies reported outcomes at multiple time points, which time point was selected for analysis, and why?

• As only one reviewer conducted the full-text screening, there is a risk of selection bias. This should be explicitly acknowledged in the limitations section. Ideally, a second reviewer should have screened at least a subset of full-text articles.

• The results section contains substantial repetition of information already presented in the tables, particularly regarding changes in depression scores from individual studies. This section could be streamlined, and the overall logical flow improved.

• The results of the risk-of-bias assessment for non-RCTs are not reported and should be included.

• Depression outcomes measured at multiple time points (e.g., post-treatment and various follow-up periods) appear to be pooled together in a single meta-analysis. Further justification is required to explain the validity of this approach. If post-treatment outcomes were available across all studies, it may have been more appropriate to use these consistently rather than combining different time points.

• The subgroup analyses address relevant factors; however, the small sample sizes within these subgroups should be clearly acknowledged when interpreting the findings.

• Given the stated focus on LMICs, there is limited discussion of LMIC-specific issues in the discussion section. It is often unclear whether cited studies originate from HICs or LMICs, and how comparisons across these contexts are being made.

• The manuscript would benefit from careful language editing. Issues include punctuation errors and sentence structure; for example, several sentences begin with “whereas” when they should instead continue from the preceding sentence.

• Greater attention is needed for reference formatting. In several instances, multiple references are listed in separate brackets rather than within a single set of brackets.

• Please ensure consistent and correct use of abbreviations and bracketing throughout the manuscript.

• Figure 1: The number of records assessed for eligibility is reported inconsistently, and some numbers appear to be repeated across boxes. Please review and correct the figure for accuracy.

# Editor report: Recommendation: The effectiveness of cognitive behavioral therapy-based interventions for depression and anxiety in people living with HIV in low- and middle-income countries: A systematic review and meta-analysis — R0/PR4

**DOI:** 10.1017/gmh.2026.10223.pr4

**Affiliations:** Psychology, UNISA, South Africa

Dear Miss Mughal

We thank you for the submission of your manuscript. The editorial and peer

Reviews of your manuscript have now been completed, and we have reached a

decision regarding your submission.

As you can see from the comments included, the reviewers recommend

major revisions to your manuscript. We strongly encourage you to

submit a revised version that addresses the reviewers’ concerns.

Should you choose to revise the manuscript, please be sure to take into

careful consideration, the suggestions of the reviewers. Please include with

your revised submission, an itemised, point-by-point response to the

reviewers, which details the changes made. 

See below the comments that need to be addressed

Reviewer 1

While thanking you for your valuable research, here are a few suggestions:

In lines 75 to 78, you did not use references in front of the sentences. It is suggested that you use the following related reference: Abdollahpour, Sedigheh, et al. “The efficacy of cognitive behavioural therapy on stress, anxiety and depression of infertile couples: a systematic review and meta-analysis.” Journal of Obstetrics and Gynaecology 42.2 (2022): 188-197.

Many other sentences in the introduction section are without references. For example, in line 103, you could use the following reference: Abdollahpour, Sedigheh, et al. “Effect of brief cognitive behavioral counseling and debriefing on the prevention of post-traumatic stress disorder in traumatic birth: a randomized clinical trial.” Community mental health journal 55.7 (2019): 1173-1178.

Abdollahpour, Sedigheh, et al. “The effect of debriefing and brief cognitive-behavioral therapy on postpartum depression in traumatic childbirth: a randomized clinical trial.” Journal of Midwifery & Reproductive Health 6.1 (2018).

In the search strategy, include the words you combined and used AND - OR

Did you not subgroup the results based on the number of counseling sessions? In the meta-analysis, were the depression questionnaires the same or different? How did you meta-analyze the results when the questionnaires were not the same?

Why did you not draw the risk of bias and forest plot?

Some of the references are old and need to be revised.

Reviewer 2

Thank you for the opportunity to review this systematic review and meta-analysis. The study addresses an important topic; however, substantial revisions are required before it can be considered for publication in this journal. Please find my comments below.

• In the introduction, the authors attempt to establish a rationale for conducting this review; however, this section could be strengthened by providing a clearer explanation of why findings from high-income countries (HICs) may not be directly transferable to low- and middle-income countries (LMICs) in this context.

• A clear definition of LMICs should be provided, including how this classification was determined.

• Line 61: There is a typographical error—“In Pakistan” should replace “n Pakistan.”

• Additional detail is required in the methods section regarding the screening and selection process. Specifically:

o Were any diagnostic criteria applied for HIV?

o Were specific questionnaires or classification systems required for the assessment of depression and/or anxiety?

o What was the rationale for including both randomized controlled trials (RCTs) and non-randomized controlled studies (non-RCTs)?

o How was LMIC status determined?

o If studies reported outcomes at multiple time points, which time point was selected for analysis, and why?

• As only one reviewer conducted the full-text screening, there is a risk of selection bias. This should be explicitly acknowledged in the limitations section. Ideally, a second reviewer should have screened at least a subset of full-text articles.

• The results section contains substantial repetition of information already presented in the tables, particularly regarding changes in depression scores from individual studies. This section could be streamlined, and the overall logical flow improved.

• The results of the risk-of-bias assessment for non-RCTs are not reported and should be included.

• Depression outcomes measured at multiple time points (e.g., post-treatment and various follow-up periods) appear to be pooled together in a single meta-analysis. Further justification is required to explain the validity of this approach. If post-treatment outcomes were available across all studies, it may have been more appropriate to use these consistently rather than combining different time points.

• The subgroup analyses address relevant factors; however, the small sample sizes within these subgroups should be clearly acknowledged when interpreting the findings.

• Given the stated focus on LMICs, there is limited discussion of LMIC-specific issues in the discussion section. It is often unclear whether cited studies originate from HICs or LMICs, and how comparisons across these contexts are being made.

• The manuscript would benefit from careful language editing. Issues include punctuation errors and sentence structure; for example, several sentences begin with “whereas” when they should instead continue from the preceding sentence.

• Greater attention is needed for reference formatting. In several instances, multiple references are listed in separate brackets rather than within a single set of brackets.

• Please ensure consistent and correct use of abbreviations and bracketing throughout the manuscript.

• Figure 1: The number of records assessed for eligibility is reported inconsistently, and some numbers appear to be repeated across boxes. Please review and correct the figure for accuracy. 

Thanks

Dr Limkile Mpofu (Handling Editor, Cambridge Prisms: Global Mental Health) 

University of South Africa (UNISA) Department of Psychology

# Editor report: Decision: The effectiveness of cognitive behavioral therapy-based interventions for depression and anxiety in people living with HIV in low- and middle-income countries: A systematic review and meta-analysis — R0/PR5

**DOI:** 10.1017/gmh.2026.10223.pr5

**Affiliations:** London School of Hygiene & Tropical Medicine, United Kingdom of Great Britain and Northern Ireland

No accompanying comment.

# Reviewer report: Review: The effectiveness of cognitive behavioral therapy-based interventions for depression and anxiety in people living with HIV in low- and middle-income countries: A systematic review and meta-analysis — R1/PR7

**DOI:** 10.1017/gmh.2026.10223.pr7

Thank you for the opportunity to re-review this manuscript. The authors have made a good effort to address the concerns raised by the reviewers, which has improved the manuscript. However, a few issues remain that should be addressed before the manuscript is accepted.

• The changes in depression scores reported for individual studies are not necessary in the text of the Results section and would be more appropriate in the table describing the included studies.

• Minor referencing errors are present. For example, in Line 284: “Three in health consultant clinics (Hemmati Sabet et al., 2013; Atefeh Nobakht et al., 2018).” The sentence refers to three studies but only includes two references. Please check the manuscript carefully to ensure that all references are correctly cited.

• Some grammatical issues remain throughout the manuscript; therefore, the authors may benefit from a language editing service. Examples include:

o Since mental health problems lie along a continuum that extends from mild distress to persistent and severe symptoms, treatment for such conditions is crucial (Patel, 2018).

o Previous systematic reviews on the use of CBT among PLHIV have largely focused on its effectiveness for depression in high-income countries (HICs), which limits their generalizability to LMIC settings and to other common conditions such as anxiety (Gebru et al., 2024).

o Some studies included follow-up periods of up to three years; however, only the post-intervention outcomes at the 12-month follow-up were used in this review (Guo et al., 2020).

• Results should not be presented in the Methods section. For example, the results of the sensitivity analysis currently described on Pg. 10 (Line 247) should instead be moved to the Results section.

Overall, the manuscript has improved following revision, and addressing the points above will further strengthen its clarity and presentation. I therefore recommend minor revision prior to acceptance. However, careful language editing throughout the manuscript is strongly recommended, as clarity and grammatical accuracy remain a notable concern.

# Editor report: Recommendation: The effectiveness of cognitive behavioral therapy-based interventions for depression and anxiety in people living with HIV in low- and middle-income countries: A systematic review and meta-analysis — R1/PR8

**DOI:** 10.1017/gmh.2026.10223.pr8

**Affiliations:** Psychology, UNISA, South Africa

Dear Miss Mughal

We thank you for working on your manuscript. The second review of your manuscript has now been completed; however, the reviewers think there is also a need to address the minor revisions to your manuscript. We strongly encourage you to submit a revised version that addresses the reviewers’ concerns.

Please take into careful consideration, the suggestions of the reviewers, especially the language editing of your manuscript. Please include with your revised submission, an itemised, point-by-point response to the reviewers, which details the changes made. 

See below the comments that need to be addressed

Reviewer 1

Thank you for the opportunity to re-review this manuscript. The authors have made a good effort to address the concerns raised by the reviewers, which has improved the manuscript. However, a few issues remain that should be addressed before the manuscript is accepted.

• The changes in depression scores reported for individual studies are not necessary in the text of the Results section and would be more appropriate in the table describing the included studies.

• Minor referencing errors are present. For example, in Line 284: “Three in health consultant clinics (Hemmati Sabet et al., 2013; Atefeh Nobakht et al., 2018).” The sentence refers to three studies but only includes two references. Please check the manuscript carefully to ensure that all references are correctly cited.

• Some grammatical issues remain throughout the manuscript; therefore, the authors may benefit from a language editing service. Examples include:

o Since mental health problems lie along a continuum that extends from mild distress to persistent and severe symptoms, treatment for such conditions is crucial (Patel, 2018).

o Previous systematic reviews on the use of CBT among PLHIV have largely focused on its effectiveness for depression in high-income countries (HICs), which limits their generalizability to LMIC settings and to other common conditions such as anxiety (Gebru et al., 2024).

o Some studies included follow-up periods of up to three years; however, only the post-intervention outcomes at the 12-month follow-up were used in this review (Guo et al., 2020).

• Results should not be presented in the Methods section. For example, the results of the sensitivity analysis currently described on Pg. 10 (Line 247) should instead be moved to the Results section.

Overall, the manuscript has improved following revision, and addressing the points above will further strengthen its clarity and presentation. I therefore recommend minor revision prior to acceptance. However, careful language editing throughout the manuscript is strongly recommended, as clarity and grammatical accuracy remain a notable concern. 

Thanks

Dr Limkile Mpofu (Handling Editor, Cambridge Prisms: Global Mental Health) 

University of South Africa (UNISA) Department of Psychology

# Editor report: Decision: The effectiveness of cognitive behavioral therapy-based interventions for depression and anxiety in people living with HIV in low- and middle-income countries: A systematic review and meta-analysis — R1/PR9

**DOI:** 10.1017/gmh.2026.10223.pr9

**Affiliations:** London School of Hygiene & Tropical Medicine, United Kingdom of Great Britain and Northern Ireland

No accompanying comment.

# Editor report: Recommendation: The effectiveness of cognitive behavioral therapy-based interventions for depression and anxiety in people living with HIV in low- and middle-income countries: A systematic review and meta-analysis — R2/PR11

**DOI:** 10.1017/gmh.2026.10223.pr11

**Affiliations:** Psychology, UNISA, South Africa

Dear Miss Mughal,

Thank you for your revised manuscript. We have reached a decision regarding

your submission. I am pleased to inform you that your manuscript has now

been accepted for publication.

The Editorial Office will contact you in due course.

Thank you for submitting your interesting and important work to Cambridge Prisms: Global Mental Health.

Kind regards,

Dr Limkile Mpofu (Handling Editor, Cambridge Prisms: Global Mental Health)

University of South Africa (UNISA) Department of Psychology

# Editor report: Decision: The effectiveness of cognitive behavioral therapy-based interventions for depression and anxiety in people living with HIV in low- and middle-income countries: A systematic review and meta-analysis — R2/PR12

**DOI:** 10.1017/gmh.2026.10223.pr12

**Affiliations:** London School of Hygiene & Tropical Medicine, United Kingdom of Great Britain and Northern Ireland

No accompanying comment.
